# Prolonged hospital stays and associated factors among hyperglycemic crisis patients admitted to public hospitals in Ethiopia: A prospective observational study

**DOI:** 10.1371/journal.pone.0342164

**Published:** 2026-02-26

**Authors:** Yadeta Babu Bayane, Yonas Abebe Abebe, Daniel Mitiku Yigazu, Fekede Bekele Daba, Ahmed Abdela Usman

**Affiliations:** 1 Department of Clinical Pharmacy, Institute of Health Science, Wallaga University, Nekemte, Ethiopia; 2 Department of Midwifery, College of Health Sciences, Dilla University, Dilla, Ethiopia; 3 Department of Clinical Pharmacy, Institute of Health Science, Jimma University, Jimma, Ethiopia; 4 Department of Clinical Pharmacy, College of Medicine and Health Science, Wachemo University, Hosanna, Ethiopia; Gabriele d'Annunzio University of Chieti and Pescara: Universita degli Studi Gabriele d'Annunzio Chieti Pescara, ITALY

## Abstract

**Background:**

A hyperglycemic crisis represents a critical metabolic emergency with potentially fatal consequences and significant health complications. These crises account for approximately 1% of hospital admissions for individuals with diabetes. While hospital admission is essential for providing the intensive care and inpatient services necessary to sustain life, prolonged hospital stays are a major risk factor for increased morbidity and in-hospital mortality among patients admitted for hyperglycemic crises. Therefore, this study aimed to determine the prevalence of prolonged hospital stays and associated factors among hyperglycemic crisis patients admitted to public hospitals in Ethiopia between July and November 2022.

**Methods:**

This research utilized a hospital-based multicenter prospective observational study design over a period of five months. Patient data were gathered through interviews and chart reviews. The duration of hospital stay was assessed by following the patients from admission to discharge. The collected data were systematically cleaned, coded, and entered into Epi Data Manager version 4.6, subsequently exported to the Statistical Package for the Social Sciences (SPSS) version 25.0. Multiple step-wise backward logistic regression was employed to identify factors influencing the length of hospital stay. The strength of associations between dependent and independent variables was examined using a 95% confidence interval (CI), adjusted odds ratio (AOR) and a p-value of less than 0.05.

**Results:**

This study included a total of 213 patients with hyperglycemic crisis. A mean age of participants was 40.37 ± 16.87 SD. About 58.2% (95% CI: 51.3–64.9%) of admitted patients were hospitalized for longer than a week, and the mean of hospital stay was 7.21 days. Age of older than 45 years [AOR:6.0,95%CI:2.27–15.93], female gender [AOR:2.76,95%CI:1.4–5.42], type 2 diabetes [AOR: 3.78,95%CI:1.36–10.48], and hypernatremia [AOR:4.31,95%CI: 1.1–16.98] were factors associated with prolonged hospital stays.

**Conclusions:**

The findings from this study underscore significant factors contributing to prolonged hospitalization in patients experiencing hyperglycemic crises. These group of patients need more emphasize and tailored management strategies to potentially mitigate the duration of hospitalizations and improve overall healthcare outcomes.

## Introduction

A hyperglycemic crisis is a severe metabolic emergency linked to poorly managed diabetes mellitus, which can lead to considerable health complications or even death [1].Diabetic ketoacidosis (DKA) and hyperglycemic hyperosmolar state (HHS) are the two most critical and potentially fatal hyperglycemic crisis affecting individuals with both type 1 diabetes (T1D) and type 2 diabetes (T2D) [2,3]. Global statistics indicate a rise in admissions for DKA and HHS over the last ten years, with recent figures highlighting a 55% increase in DKA hospitalizations, particularly among adults under 45 years old [4,5].

Hyperglycemic crises represent a primary determinant of adverse outcomes associated with diabetes, including mortality, morbidity, and substantial healthcare expenditure. Recent epidemiological analyses highlight an alarming trend: a 35% increase in hospital admissions for diabetic crises occurred between 1996 and 2006, emphasizing the growing burden of this condition [3,6]. A United States-based study indicates that 38% of hospital admissions for hyperglycemic crises were attributed to diabetic ketoacidosis (DKA), 35% to hyperglycemic hyperosmolar state (HHS), and 27% to concurrent DKA and HHS [7].

DKA leads to more than 500,000 hospital days with an estimated direct and indirect medical cost of $2.4 billion each year [8,9]. Hyperglycemic crises represent a significant burden on healthcare systems in Africa, particularly for diabetic patients. Hospital admissions due to these crises range from approximately 26% in South Africa to 40% in Nigeria. The consequences are dire, as evidenced by mortality rates of 7.5% and 34% in South Africa and Nigeria, respectively [10,11]. In Ethiopia, adult medical admissions and the expense of inpatient diabetes care, including medication use and bed occupancy, are significantly influenced by hyperglycemic crisis [12].

Length of hospital stay (LOHS) is a key performance indicator for hospitals, representing the time elapsed between a patient's admission and discharge [13,14].A prolonged length of stay (LOS) occurs when a patient's hospitalization exceeds the expected duration for their specific treatment. This can signal complications, inefficient care processes, or other issues delaying discharge [15,16]. In the year 2012, only 2% of patients experienced hospital stays that exceeded 21 days. Presently, however, this group represents 14% of all hospital days and incurs an annual expenditure exceeding $20 billion [17].

In European healthcare facilities, the incidence of prolonged hospital stays among patients ranges from 7.98% to 16.67% [18,19]. Research conducted in some part of Asian countries indicates that a significant portion of patients, ranging from 5.4% to 30.5%, experienced extended durations of hospitalization [20,21].In various regions across Africa, including Ethiopia, it has been observed that between 24.7% and 63% of patients endure extended durations of hospitalization [22,23].

Globally, studies indicate several factors correlate with extended hospitalizations for patients experiencing hyperglycemic crises. These include elevated total serum osmolality, advanced age, female gender, absence of a pre-existing diagnosis of insulin-dependent diabetes, compromised mental acuity upon admission, diminished serum potassium, pH, and bicarbonate (HCO3) levels [24,25]. Further investigation into these elements may lead to improved patient outcomes and reduced hospital stays. To the best of our understanding, there are no prior investigations that have explored the factors contributing to extended hospital stays among patients experiencing hyperglycemic crisis in Ethiopia. Consequently, our study aims to identify the incidence of prolonged hospitalization and the associated factors among hyperglycemic crisis patients admitted to public hospitals in Ethiopia.

## Materials and methods

### Study design and study setting

A prospective observational study was conducted between July 1 and November 30, 2022, to investigate adolescent and adult diabetic patients admitted with hyperglycemic crisis. Participants were recruited from the emergency, medical, and pediatric wards of three Ethiopian hospitals: Adama Hospital and Medical College (AHMC), Jimma Medical Center (JMC), and Shanen Gibe General Hospital (SGH). JMC, situated in Jimma town, approximately 352 km southwest of Addis Ababa, is a major teaching institution and the sole teaching and referral hospital in southwestern Ethiopia, serving a catchment population of around 15 million. AHMC, located in Adama town, East Showa Zone, 99 km from Addis Ababa, provides inpatient services with over 200 beds to a catchment population of 4,690,000. SGH is located in southwestern Ethiopia and delivers inpatient and outpatient services to an estimated population of 1,209,60.

### Study population and eligibility criteria

The selection of participants for this study focused on adolescent and adult patients with diabetes mellitus (DM) who presented with hyperglycemic crisis at the emergency, medical, and pediatric wards of JMC, AHMC, and SGH during the designated study period. To ensure data integrity and relevance, strict eligibility criteria were applied. Participants were required to be at least 13 years of age, possess a confirmed diagnosis of hyperglycemic crisis, and have complete medical records available for review. Conversely, patients who were refused to give consent, admitted to wards outside the specified departments, died during hospitalization, and transferred to the intensive care unit (ICU) were excluded from the study.

### Sample size determination and sampling technique

**Fig 1:**
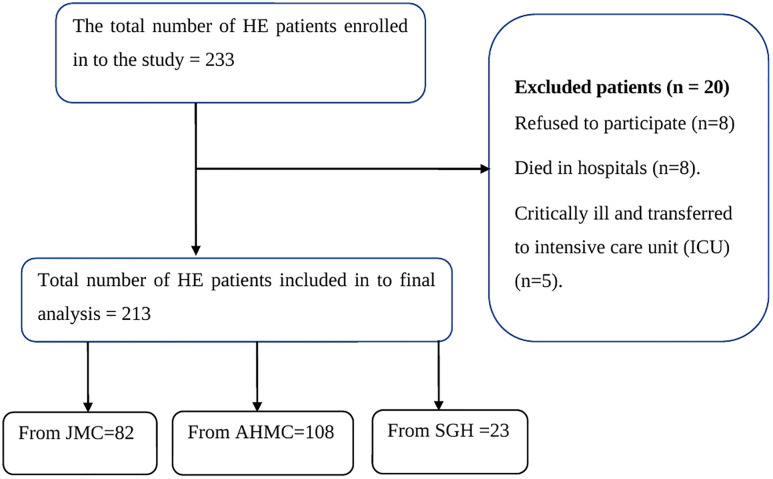
Patients enrollement in to HE at selected Ethiopian hospitals from July to Novomber 2022.

Over a five-month period, this study consecutively enrolled 233 patients. Sample size was determined using a single population proportion formula, incorporating the following parameters: a Z-score of 1.96, an estimated proportion (P) of 0.5%, a 95% confidence level, a 5% margin of error. By considering the above assumptions initial sample size (n) of 384 was determined. To refine the sample size, a reduction formula was applied, utilizing data indicating a total of 473 hyperglycemic crisis patients across three hospitals (SGH, JMC, and AHMC) during the preceding five months, nf = n / (1 + n/N), the adjusted sample size (nf) was calculated to be 212. Accounting for a 10% non-response rate, the final required sample size was increased to 233. This sample was proportionally distributed among the three hospitals: JMC, AHMC, and SGH (Fig 1).

### Data collection procedure

The data collection process commenced after the patient stabilized in the emergency department. Consent was taken and eligible patients were assessed for various pertinent information. Recorded information in the data abstraction tools include: day of admission, day of discharge, patient identification number (ID), socio demographic details, clinical characteristics, laboratory results, medication-related factors, and disease-related aspects. These information was systematically extracted using a designated data abstraction tools. Data collectors, consisting of three first-degree nurses, strictly followed patients starting from day of admission to discharge including who were subsequently transferred to either medical or paediatrics wards by using the given patient identification number (ID). Additionally, two clinical pharmacists oversaw the entire process to ensure accuracy and consistency in data collection process.

### Model diagnostic test

The Hosmer-Lemeshow test was employed to assess the goodness-of-fit of the model. The resulting p-value of 0.667 exceeds the conventional significance level of 0.05. Therefore, based on the Hosmer-Lemeshow test, there is no statistically significant evidence to reject the null hypothesis, suggesting that the model exhibits an acceptable fit to the data. To ensure the robustness of the statistical analysis, multicollinearity among the independent variables was rigorously assessed. This assessment employed variance inflation factor (VIF) and tolerance as diagnostic measures. The results of these tests indicated that no significant multicollinearity was present within the datasets.

### Data quality assurance

Before initiating the actual data collection, a thorough assessment of the questionnaire was conducted to ensure its completeness. A comprehensive training session lasting one full day was provided for data collectors and supervisors to familiarize them with the data abstraction tool. Additionally, a pre-test was administered involving 5% of the required sample size, leading to minor adjustments based on the feedback obtained from this preliminary test. Throughout the data collection process, supervision was maintained, and the principal investigator (PI) conducted daily reviews of the collected data to ensure accuracy and reliability.

### Statistical analysis

The gathered data underwent a thorough process of compilation, cleaning, coding, and verification for completeness and accuracy prior to being entered into the respective software applications. The EpiData Manager, version 4.6, was utilized for data entry, while statistical analyses were conducted using SPSS version 25.0. All statistical evaluations were carried out within the SPSS environment. Descriptive statistics, including frequencies, means, and standard deviations (SD), were calculated to summarize the data. To explore the relationship between dependent and independent variables, a univariable logistic regression analysis was undertaken. Independent variables exhibiting a p-value of less than 0.25 were subsequently incorporated into a final multivariate model to identify determinants of prolonged hospital stay. In all statistical evaluations, a p-value of less than 0.05 was established as the threshold for statistical significance. To quantify the strength of association between the independent and dependent variables, odds ratios with corresponding 95% confidence intervals (CI) were computed. The results of the data processing were effectively communicated through the use of tables and figures, facilitating a clear understanding of the analytical outcomes.

### Ethical consideration

Ethical clearance and an approval letter were secured from the Institutional Review Board (IRB) of Jimma University, referenced as IRB No. IHRPGY/1203/2022. The hospital director, along with the heads of departments, including those in the emergency, medical, and pediatric wards, were informed about the study's objectives and asked to provide their support. Prior to the commencement of the research, written informed consent was obtained from all participants. A paramount concern throughout the process was the confidentiality of patient data; therefore, we utilized patient identification numbers instead of names to protect individual identities. Furthermore, the information collected was strictly limited to the principal investigator and was stored in a locked cabinet to ensure privacy and prevent unauthorized access.

### Definition of terms

**Hyperglycemic crisis:** is extreme metabolic derangements including DKA and HHS associated with uncontrolled types 1 and 2 diabetes mellitus [1,3].

**DKA:** is an acute, major, life-threatening complication of diabetes characterized by hyperglycemia, ketoacidosis, and ketonuria [26,27].

**HHS:** is characterized by hyperglycemia, hyperosmolarity, and dehydration, without significant ketoacidosis [28,29]

**Prolonged hospital stays:** is a hospital stay for more than seven days starting from day of admission [10,30].

## Results and discussion

### Sociodemographic characteristics of study participants at public hospitals in Ethiopia

This study included a total of 213 hyperglycemic emergency patients. A higher proportion of participants were female (62.45%) compared to male (37.55%). Furthermore, the sample population tended towards older age ranges (>= 45 years). In terms of geographic distribution, a majority (≈ 60%) of participants resided in urban areas. Regarding educational attainment, a substantial portion (38.49%) had completed primary education (Table 1).

**Table 1:** Demographic distributions of study participants at public hospitals in Ethiopia, 2022.

Variables	Responses	Frequency (n)	Percentage (%)
Age in years	13-28	68	31.92
	29-44	66	30.98
	>=45	79	37.08
Gender	Male	80	37.55
	Female	133	62.45
Ethnicity	Oromo	109	51.17
	Amhara	60	28.16
	Others	44	20.65
Religion	Muslims	94	44.13
	Orthodox	82	38.49
	Protestants	37	17.37
Marital status	Married	146	68.54
	Single	60	28.16
	Others	7	3.28
Occupation	Farmer	69	32.39
	Merchants	39	18.30
	Employees	34	15.96
	Students	30	14.08
	Daily laborer	41	19.24
Residence	Urban	127	59.62
	Rural	86	40.37
Education	No formal education	39	18.30
	Primary education	82	38.49
	Secondary education	56	26.29
	College and above	36	16.90

### Clinical characteristics of hyperglycemic crisis patients admitted to public hospitals in Ethiopia

A prospective analysis of 213 patients revealed that diabetic ketoacidosis (DKA) was the primary cause of admission in a significant majority (70.42%), while hyperosmolar hyperglycemic state (HHS) accounted for the remaining 29.58%. Comorbidities were present in over a quarter of the patients (25.36%), with hypertension and chronic heart failure being the most prevalent. In-hospital complications included hypoglycemia (25.35%), hypokalemia (15.96%), and hypernatremia (13.61%). Notably, a substantial proportion of patients, 58.2% (95% CI: 51.3–64.9%), required extended hospitalization exceeding seven days (Table 2).

**Table 2:** Clinical Characteristics of hyperglycemic crisis patients admitted to the public hospitals in Ethiopia, 2022.

Characteristics	Responses	Frequency (n)	Percentage (%)
Parental diabetes	Yes	49	23.00
	No	164	76.99
Types of diabetes	Type 1	129	60.56
	Type 2	84	39.43
Acute complications	DKA^a^	150	70.42
	HHS^b^	63	29.58
Previous admission	Yes	55	25.82
	No	158	74.17
Comorbidity	Yes	54	25.36
	No	159	74.64
Types of comorbidities	Hypertension	17	7.98
	CHF^c^	12	5.63
	CKD^d^	10	4.69
	Pulmonary disease	7	3.28
	Others^*^	8	3.75
Drug discontinuations	Yes	86	40.37
	No	127	59.62
Hypoglycemia	Yes	54	25.35
	No	159	74.64
Hypernatremia	Yes	29	13.61
	No	184	86.38
Hypokalemia	Yes	34	15.96
	No	179	84.03
GCS^e^	< 8	43	20.18
	9-15	170	79.81
Hospitalization	<= 7 days	89	41.78
	>7days	124	58.21

^a^: Diabetic ketoacidoisis, ^b^: Hyperosmolar hyperglycemic state, ^c^: Chronic heart failure, ^d^: Chronic kidney disease, ^e^: Glascow coma score.

### Factors associated with prolonged hospital stay among patients with hyperglycemic crisis

Univariate, and multivariate logistic regression analyses were conducted to identify the factors influencing duration of hospital stays. From the univariate analysis, variables with a P-value of less than 0.25 were selected for further evaluation in the multivariable analysis. Based on the results of the univariate analysis, several candidate variables emerged for further scrutiny. These included age over 45 years, female gender, secondary education, type 2 diabetes, previous admission, hypernatremia, hypokalemia, and a Glasgow Coma Scale (GCS) score of less than 8. Further examination of the candidate variables revealed that patients aged 45 years or older (AOR: 6.0, P < 0.01, CI: 2.27–15.93), female gender (AOR: 2.76, P < 0.01, CI:1.40–5.42), type 2 diabetes (AOR: 3.78, P = 0.010, CI:1.36–1.48) and hypernatremia (AOR: 4.31, P = 0.036, CI:1.1–16.98) were significantly correlated with prolonged hospital stays (Table 3).

**Table 3:** Results of univariate and multivariate analysis of factor affecting prolonged hospital stays among hyperglycemic crisis patients admitted to public hospitals in Ethiopia.

Characteristics	Responses	COR^g^	P-Value	95%CI	AOR	P-Value	95%CI
Age in years	13-28	Ref			Ref		
	29-44	1.85	0.079	0.93-3.71	2.47	0.061	1.48-4.11
	>= 45	1.73	0.102	0.89-3.34	6.0	**< 0.01**	2.27-15.93
Gender	Male	Ref			Ref		
	Female	2.6	< 0.01	1.47-4.60	2.76	**< 0.01**	1.40-5.42
Residence	Urban	Ref			Ref		
	Rural	1.16	0.584	0.66-2.03	----	----	----
Educational status	No formal	0.85	0.725	0.34-2.10	0.76	0.630	0.25-2.31
	Primary	1.20	0.648	0.54-2.64	1.06	0.893	0.41-2.73
	Secondary	2.05	0.104	0.86-4.88	2.46	0.086	0.88-6.88
	College	Ref	----	----	Ref	----	----
Occupation	Farmer	0.64	0.278	0.29-1.41			
	Merchant	3.89	0.313	1.33-11.34			
	Daily worker	0.71	0.460	0.28-1.76			
	Student	0.92	0.875	0.35-2.40			
	Employee	Ref	----	----			
Parental diabetes	Yes	1.05	0.862	0.55-2.01			
	No	Ref	----	----			
Types of diabetes	Type 1	Ref			Ref		
	Type 2	2.86	< 0.01	1.62-5.05	3.78	**0.010**	1.36-1.48
Acute complication	DKA	1.04	0.830	0.57-1.91			
	HHS	Ref					
Previous admission	Yes	1.64	0.113	0.88-3.06	0.51	0.072	0.24-1.06
	No	Ref			Ref		
Comorbidity	Yes	1.30	0.414	0.69-2.46			
	No	Ref			Ref		
Drug discontinued	Yes	1.26	0.406	0.72-2.21			
	No	Ref					
Hypernatremia	Yes	7.60	< 0.01	2.22-26.01	4.31	**0.036**	1.1-16.98
	No	Ref			Ref		
Hypokalemia	Yes	2.24	0.052	0.99-5.081	1.55	0.387	0.57-4.25
	No	Ref			Ref		
GCS	< 8	2.86	0.007	1.32-6.18	2.26	0.085	0.89-5.73
	9-15	Ref			Ref		

^g^: Crude odds ratio.

## Discussion

Diabetic individuals face a heightened risk of complications such as infections, impaired wound healing, and vascular damage. These risks can be significantly elevated during extended hospitalizations, primarily due to reduced physical mobility and possible interruptions to their standard diabetes management protocols. Such factors can complicate their overall health status, underscoring the importance of vigilant care and carefully managed environments for diabetic patients in hospital settings [31–33].

In this study, we found that a significant proportion of patients with hyperglycemic crisis remained hospitalized for more than a week, amounting to 58.2%(95%CI:51.3–64.9%). This rate is considerably higher than the corresponding figures from studies conducted at Jimma Medical Center (47%) [34], and Debretabor General Hospital (20.41%) [35]. Variations in clinical characteristics and, methodological differences in the study design offer a significant explanation for the divergent findings. Specifically, the earlier studies focused exclusively on diabetic ketoacidosis (DKA) patients and employed a retrospective data collection approach. In contrast, the current prospective study encompassed both DKA and hyperglycemic hyperosmolar state (HHS), a condition often associated with more severe metabolic derangement and potentially longer recovery periods. The inclusion of HHS patients likely contributed to the higher overall rate of prolonged hospital stays observed in the current study. Furthermore, the prospective design allows for more complete and accurate capture of hospitalization duration compared to retrospective chart reviews, which may be subject to data omissions or inaccuracies, potentially underestimating the true length of stay in the previous studies.

Research consistently indicates a positive correlation between age, and the duration of hospital stays for diabetic patients. Specifically, this means that as diabetic patients age, their average hospital stays tend to be longer [32,36,37].In this study, our findings indicate that diabetic patients aged 45 years or older are six times (AOR: 6, 95%CI: 2.27–15.93) more likely to be hospitalized for durations exceeding one week. This observation can be explained by the fact that, as individuals age, they are increasingly susceptible to complications associated with diabetes. These complications may include cardiovascular disease, kidney disease, and nerve damage, all of which can contribute to a higher frequency of hospitalizations and extended lengths of stay.

The duration of hospital stays exhibits a complex relationship with gender. While existing literature presents conflicting evidence, with some studies suggesting longer stays for men and others for women, our study reveals a significant association between female gender and extended hospitalization. Specifically, female patients were 2.76 times (AOR: 2.76, 95%CI: 1.40–5.42) more likely to remain hospitalized for over a week compared to their male counterparts. This observation aligns with prior research indicating that women may experience a 12% increase in length of stay and a 26% higher risk of readmission compared to males [38]. Several factors could explain this disparity, including variations in disease presentation, differences in access to healthcare, and potential delays in diagnosis. However, it is important to acknowledge that studies conducted in Australia [39] and the USA [40] have reported contradictory findings, highlighting the need for further investigation to fully understand the interplay between gender and hospital stay duration.

When examining the relationship between different types of diabetes and the duration of hospital stays, it is generally observed that patients with type 2 diabetes experience longer hospitalizations compared to those with type 1 diabetes as indicated in the data reported from Ethiopia, USA, and Romania [38,41,42]. In this context, our research revealed that patients with type 2 diabetes experienced prolonged hospitalization 3.78 times (AOR: 3.78, 95%CI: 1.36–1.48) than those of patients with type 1 diabetes. This disparity can largely be attributed to the increased prevalence of complications associated with poorly controlled type 2 diabetes. Such complications frequently lead to more frequent and severe hospital admissions, resulting in extended periods of hospitalization.

In contrast to the our study, a retrospective cross-sectional analysis of all DKA admissions from 2015 to 2021 across four hospitals in Qatar showed that hyponatremia was a predictor for length of hospital stay [43].In this study, hypernatremia was observed in 13.6% of the patients, and the risk of prolonged hospitalization was found to be 4.31 times greater in this cohort compared to the reference group. The disparity of the finding may be related with target group of the study, as the prior study included only DKA patients in which hyponatremia is more common than hypernatremia. Hypernatremia in patients with Diabetic Ketoacidosis (DKA),and Hyperglycemic Hyperosmolar State (HHS) usually arises from significant dehydration, and insufficient water consumption [44,45]. It is associated with increased morbidity, higher in-hospital mortality, and is a predictor of a prolonged length of hospital stay (LOS) [46]. This significant association highlights the impact of hypernatremia on patient outcomes and underscores the necessity for vigilant monitoring and management of sodium levels in clinical settings.

## Conclusions

A considerable number of patients were found to have hospital stays exceeding one week. The study identified a significant correlation between prolonged hospitalization and several factors, including advanced age, female gender, type 2 diabetes, and hypernatremia. To reduce mortality rates and improve the quality of hospital care, it is crucial to prioritize the management and monitoring of patients exhibiting these risk factors.

### Limitations

The present study, while providing potentially valuable insights, possesses several limitations that require careful consideration. The small sample size restricts the generalizability of findings to a larger population. The study's short duration further limits the scope of conclusions; as longitudinal studies are typically needed to fully understand long-term effects. The exclusion of ICU patients and patients who died in the hospital may influence the results, potentially underestimating the overall length of hospital stays. Furthermore, the absence of key variables such as serum osmolality, pH, and bicarbonate, due to infrequent and inconsistent measurement, further constrains the analysis. Consequently, these findings offer a preliminary understanding, and further research employing larger samples and extended timelines is essential to validate and expand upon these initial observations.

## Supporting information

S1 File Data Set. (SAV)

S2 File Data abstraction tools. (DOCX)
