# Supplementary material for: Prolonged hospital stays and associated factors among hyperglycemic crisis patients admitted to public hospitals in Ethiopia: A prospective observational study
Source: PLoS One. 2026 Feb 26;21(2):e0342164. doi: 10.1371/journal.pone.0342164 (PMC12944714; doi:10.1371/journal.pone.0342164)
Supplement: S2 File — (DOCX) [file pone.0342164.s002.docx]

ANNEX I: QUESTIONNAIRE

JIMMA UNIVERSITY

INISTITUTE OF HEALTH SCIENCE

DEPARTMENT OF CLINICAL PHARMACY

Questionnaire:-Data collection format for research paper entitled with “***prolonged hospital stay and associated factors among patients diagnosed with hyperglycaemic crisis at selected hospitals, Ethiopia from July to November 2022”.***

**PART I: Socio demographic characteristic of the study participants.**

1. Patient identification number:-----------------
2. Admission date:---------------------------------
3. Discharge date:----------------------------------
4. Age in years:-------------
5. Gender: a) Male b) Female
6. Ethnicity: a) Oromo b) Amhara c) Gurage d) Somali e) Tigre f) Others: specify
7. Religion: a) Muslim b) Orthodox c) Protestant d) others specify:--------------
8. Marital status: (a) Married (b) Single (c) Divorced (d) Widowed
9. Occupation: (a) government employee (b) farmer (c) Student (d) Businessman/woman (e) Daily labor (f) house wife (g) Othersspecify:------------------
10. Place of residence: (a) urban (b) rural
11. Level of education: (a) Not educated (b) Primary (1-8) (C) Secondary (8-12) (d) Colleges and university

**PART I I: Disease and clinical related factors**

1) Family history of DM: a) Yes b) No

2) Type of DM: a) Type 1 DM b) Type 2 DM

3) Type of acute complication: a) DKA b) HHS

4) How long have you been with DM (years)? a) ≤ 5years b) 6-10 years c) >10 years

5) History of admission in the last one year: a) Yes b) No

6) Comorbidity: a) Yes b) No

7) If question number 6 is yes, please specify types of comorbidity:-----------------------

a) Hypertension b) Congestive heart failure c) Chronic kidney disease d) Chronic liver disease e) Pulmonary disease f) other specify:--------------------------

8) History of drug discontinuation; a) Yes b) No

9) Hypoglycaemia: a) Yes b) No

10) Hypokalaemia: a) Yes b) No

11) Hypernatremia: a) Yes b) No

10) Admission GCS:-------------------------
